# Associations of domestic hard water metrics with the risk of gout incidence and recurrence

**DOI:** 10.1371/journal.pone.0326052

**Published:** 2025-07-14

**Authors:** Sitong Wei, Jie Zhang, Shaoguang Ren, Dongqing Ye, Xinyu Fang

**Affiliations:** 1 Department of Epidemiology and Biostatistics, School of Public Health, Anhui Medical University, Hefei, Anhui, China; 2 Inflammation and Immune Mediated Diseases Laboratory of Anhui Province, Hefei, Anhui, China; 3 School of Public Health, Anhui University of Science and Technology, Hefei, Anhui, China; International University of Health and Welfare, School of Medicine, JAPAN

## Abstract

**Background:**

Accumulating evidence suggests that domestic hard water is linked to health outcomes, but whether there is a potential association with gout is unknown.To examine the association between domestic hard water and gout incidence and recurrence in adults aged 40–69 years from the UK Biobank.

**Methods:**

We analyzed a cohort of 448,510 participants free of gout at baseline (2006–2010) for incidence analysis and 7,231 participants with prevalent gout for recurrence. Domestic water hardness data were obtained from local water supply companies across England, Wales and Scotland in 2005 and 2013. Cox proportional hazard models were used to assess the association between water hardness and both gout incidence and recurrence. Additionally, the Cochran Armitage test was used to examine the linear trend and restricted cubic splines assessing nonlinear relationships.

**Results:**

During a median follow-up of 13.29 years, 6,521 incident events were recorded, and at a median 12.40 years, 519 gout recurrence events were identified. For incidence, compared with individuals exposed to 0–60 mg/L, the HRs (95% CIs) for the incidence of gout in the other three grades were 1.12 (1.05–1.19), 1.16 (1.05–1.29) and 1.18 (1.11–1.25), respectively. Each additional 50 mg/L of CaCO_3_, Ca and Mg increased gout risk (HRs [95% CIs] were 1.04 [1.03–1.05], 1.17 [1.13-1.20] and 1.99 [1.46–2.71], respectively). In addition, CaCO_3_, Ca and Mg demonstrated the nonlinear relationship with gout incidence (all p for nonlinearity<0.05). For recurrence, each additional 50 mg/L of Mg increased gout recurrence risk (HRs [95% CIs] was 2.97 [1.11–7.97]). And linear trend test shown was significant for Mg (P for trend = 0.03026).

**Conclusions:**

The results revealed that exposure to hard water characterized by higher concentration levels of CaCO_3_, Ca and Mg might increase the risk of gout incidence. Moreover, individuals who are subjected to higher Mg concentrations might increase the risk of gout recurrence.

## Introduction

Gout is a chronic inflammatory disease which caused by the deposition of monosodium urate crystals in both joint and non-joint structures [1]. In 2020, it affected 55.8 million people globally, marking an increase of 22.5% since 1990, with projections indicating a continuing rise in prevalence [2]. Studies have reported that the pathogenesis of gout is related to metabolic, genetic and immune factors [3–5], while environmental factors also play an important role [6].

Hard water, characterized by high mineral content (particularly CaCO_3_, Ca, and Mg [7]), was found to be associated with various health outcomes [8–10]. Currently, no epidemiological studies investigate the association between hard water and the incidence or recurrence of gout, but previous mechanistic research provides some clues. Elevated Ca concentrations can lead to increased urinary Ca and the saturation of Ca oxalate, resulting in crystal blockage of renal tubules. Such blockages may cause renal tubule interstitial rupture and lead to the conversion of xanthine dehydrogenase into xanthine oxidase, which promotes an increase in uric acid levels [11]. Additionally, Mg has been confirmed to be negatively associated with C-reactive protein (CPR) levels, a sensitive biomarker of inflammation. Mg levels also can impact oxidative stress, potentially inducing DNA damage and the release of purine nucleotides, whose catabolism ultimately gives rise to the production of urate [12].

Furthermore, studies have found a positive correlation between the Mg content in regional water supplies and the composition of urinary stones in patients from those region [9]. Specifically, in the south and east of England, areas with widely distributed hard water, there is a higher incidence of kidney stones compared to other regions [10]. Considering that both kidney stones and gout involve crystal deposition, these regional patterns suggest that mineral content in water may influence the incidence of gout.

Given that the UK is a country where hard water is widely distributed [13] and that little research attention has been paid to the association between minerals in water and gout, we utilized data from the UK Biobank to explore the relationship between concentrations of CaCO_3_, Ca and Mg in water and the incidence and recurrence of gout. Additional sensitivity analyses were conducted to assess the robustness of our findings, aiming to elucidate the potential impact of hard water on gout.

## Materials and methods

### Data source

UK Biobank is a cohort study in which recruited 502,359 participants, aged 40–69 years, from across the UK between 2006 and 2010. Participants were assigned to the nearest assessment center based on their home address for baseline data collection, including sociodemographic characteristics, lifestyle, environmental factors, and other relevant details [14].

**Fig 1:**
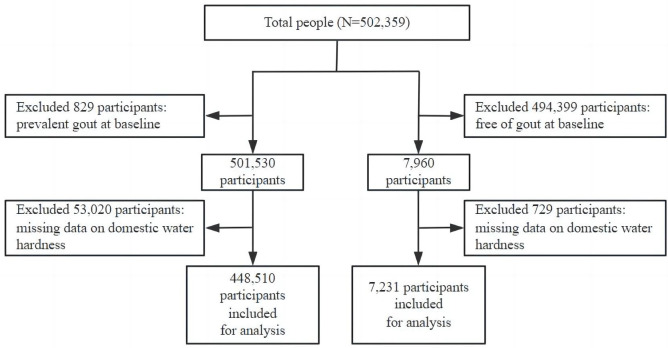
The flow charts of seeking gout incident and recurrent patients.

For the present study, in assessing the association between domestic water metrics and the incidence of gout, we excluded participants 1) who had gout at baseline (n = 829), and 2) without data for Ca and Mg concentration (n = 53,020). Finally, a total of 448,510 participants remained for the main analysis. In assessing the association between domestic water metrics and the recurrence of gout, we excluded participants 1) who were free of gout (n = 494,399), and 2) without data for Ca and Mg concentration (n = 729). Finally, a total of 7,231 participants remained for the main analysis. See Fig 1.

### Domestic hard water

Domestic water data was obtained from local water supply companies across England, Wales and Scotland. These data, collected in 2005 and 2013, include concentrations of CaCO_3_, Ca and Mg. Participants were allotted postcodes based on their reported residential addresses at baseline, which were then connected to their respective water companies. The classification of domestic hard water metrics in our study can be found in S1 Table.

### Gout definition

Gout patients were determined based on ICD-10 codes(M10.0, M10.1, M10.2, M10.3, M10.4, M10.9) and self-report (code: 1466) along with medicine-used history (codes: 1140875408, 1140909890) from hospital admission records, which containing admission diagnosis data, morbidity record data and patient event databases from England, Scotland and Wales, respectively. Participants treated with drugs for lymphoma or leukemia; those who self-reported gout but had UA levels less than 6 mg/dL, or those who did not report a history of medication were excluded. Gout recurrence was defined as having diagnostic data prior to enrollment and a recurrence after baseline (after 2010), with at least 28 days apart [15]. The follow-up period spanned from the date of initial recruitment to the onset of gout, death, loss of follow-up, or until 31 October 2022.

### Assessment of covariates

Based on previous research, several covariates were selected [7], including age (<65year, ≥ 65year), gender (female, male), ethnicity (white, other), education levels (university degree, other), income (<£18,000, £18,000–£29,999, £30,000–£51,999, £52,000–£100,000, > £100,000), BMI (kg/m^2^, continuous), smoking status (current, previous, never), drinking status (yes, no), and water intake (glasses/day, continuous), collected via a touch-screen questionnaire. The Index of Multiple Deprivation (IMD) and Townsend Index assess the extent of regional deprivation and are calculated based on national census data from before participants joined the UK Biobank to rank and compare poverty across regions. To achieve consistent score analysis across different countries, we used IMD scores adjusted for the UK [7]. Enzymatic was measured in serum urate levels which were collected at recruitment, and measured by uricase PAP analysis on a Beckman Coulter AU5800. Details of laboratory measurements are available online (https://biobank.ndph.ox.ac.uk/showcase/field.cgi?id=30880). The diet scores was calculated by vegetables and fresh fruit, fish, processed meat and unprocessed red meat [16]. Alanine aminotransferase (ALT), aspartate aminotransferase (AST), alkaline phosphatase (ALP) and γ-glutamyltransferase (GGT) were used to evaluate liver function [17]. We estimating glomerular filtration rate (eGFR) from creatinine and cystatin C [18]. In our study, the Polygenic Risk Score (PRS) was a continuous variable. The methods of genotyping and quality control in the UK Biobank have been detailed in earlier studies [19]. We accessed the standard GRS for gout released by the UK Biobank [20]. The GRS algorithms were developed from trait-specific meta-analyses utilizing a Bayesian approach that integrated data from multiple ancestries and related traits when applicable. In contrast to general GRS, which are constructed based on reported top SNPs, the per-individual GRS value is determined as the genome-wide sum of the per-variant posterior effect sizes, adjusted for allele dosage.

### Statistical analysis

Based on whether participants had an incident or recurrent gout, we summarized their baseline characteristics, where continuous variables were represented by mean (standard deviation [SD]) and categorical variables were represented by n(%).

Cox proportional hazards models were used to assess the association between hard water metrics and both gout incidence and recurrence. The association between standardized Schoenfeld residuals and time was used to evaluate the validity of the proportionality of hazards [21]. The CaCO_3_, Ca, and Mg concentrations were analyzed as continuous variables, the unit was 50 mg/L. The WHO classification, USGS classification, and Ca and Mg concentrations were used as categorical variables, with the lowest group used as the reference. The analysis utilized a minimally adjusted Cox model including age, gender and BMI as covariates (Model 1), Model 2 was with additional adjustment of ethnicity, education levels, Townsend deprivation index, IMD, income, BMI, smoking and drinking status, urate, water intake, ALT, AST, ALP, GGT, PRS and eGFR. Hazard ratios (HRs) and 95% confidence intervals (CIs) were used to evaluate the risk associated with hard water metrics in both gout incidence and recurrence. Cochran_Armitage test was used to examine the linear trend. Given the substantial disparity in sample sizes between the first (497,512 participants collected data in the 2005) and second rounds (20,137 participants collected data in the 2013), primary analyses were conducted using the first round data, with the second round data serving as a supplementary analysis (as Lopez et al.) [7]. We stratified the analyses using the two factors: age (<65 years or ≥65 years), gender (male or female) and BMI (<25 kg/m^2^ or ≥25 kg/m^2^). Interaction analyses were utilized to identify potential effect modifications.

Several sensitivity analyses were used to verify the robustness of the results based on Model 2. First, to avoid reverse causation, we limited the event cases to more than two years of follow-up. Second, to assess the stability of the link over a longer period of time, we excluded patients who developed gout within 5 years. Finally, given that hypertension and diabetes affect metabolism, we additionally adjusted for both based on Model 2. In addition, restricted cubic spline analysis with 3 knots was used to assessed the exposure-response relationship of the hard water metrics with both gout incidence and recurrence risk.

The R package “mice.” was used to perform multiple imputations for the missing covariates [22]. The numbers and percentages of participants with missing covariates are shown in S2 Table. All statistical analyses were performed using R software (Version 4.4.0). The statistical significance level was set to <0.05.

## Results

### Baseline characteristics

During 13.29 years of follow-up, we observed 6,521 gout events among 448,510 participants who had domestic hard water data. The proportion of people aged 65 years and older with gout was significantly higher than those without gout (34.6% vs. 18.7%). The average concentration of CaCO_3_, Ca, and Mg among those without gout (441,989 participants) were 130.56 mg/L, 51.07 mg/L, and 4.58 mg/L, respectively. Among the 6,521 participants with gout, these values were 127.69 mg/L, 51.20 mg/L, and 4.60 mg/L, respectively. Compared with participants without gout, the individuals with gout had lower income levels (<£18,000: 34.6% vs. 24.8%), and they were more likely to be male (77.3% vs. 44.3%) and had a higher BMI (30.34 kg/m^2^ vs. 27.35 kg/m^2^).

Among 7,231 gout patients, 519 recurrent cases of gout were recorded during 12.40 years of follow-up. The average concentrations of CaCO_3_, Ca, and Mg in 6,712 participants without gout recurrence were 124.36 mg/L, 50.60 mg/L, and 4.64 mg/L, respectively; and the 519 with recurrent gout, these values were 122.15 mg/L, 50.91 mg/L, and 4.89 mg/L, respectively. Compared with participants without gout recurrence, the individuals with recurrence had higher BMI (31.36 kg/m^2^ vs. 30.71 kg/m^2^), were older (34.3% vs. 30.1%), and were more likely to be previous or current smokers (60.9% vs. 57.8%) (Table 1).

**Table 1:** Baseline characteristics of the study samples.

	Incident gout			Recurrent gout		
Characteristics	No (n = 441989)	Yes (n = 6521)	*P*	No (n = 6712)	Yes (n = 519)	*P*
Age, [n(%])			<0.001			0.05
<65 year	359117 (81.3)	4267 (65.4)		4693 (69.9)	341 (65.7)	
≥65 year	82872 (18.7)	2254 (34.6)		2019 (30.1)	178 (34.3)	
Gender, [n(%)]			<0.001			0.874
Female	246350 (55.7)	1479 (22.7)		536 (8.0)	43 (8.3)	
Male	195639 (44.3)	5042 (77.3)		6176 (92.0)	476 (91.7)	
Ethnic, [n(%)]			0.338			0.022
White	419330 (94.9)	6169 (94.6)		6424 (95.7)	485 (93.4)	
Others	22659 (5.1)	352 (5.4)		288 (4.3)	34 (6.6)	
Education levels, [n(%)]			<0.001			0.286
University degree	168215 (38.1)	1999 (30.7)		4535 (67.6)	363 (69.9)	
Others	273774 (61.9)	4522 (69.3)		2177 (32.4)	156 (30.1)	
BMI [mean (SD)]	27.35 (4.77)	30.34 (5.14)	<0.001	30.71 (4.99)	31.36 (5.32)	0.004
Follow time [mean (SD)]	13.35 (2.03)	9.67 (3.27)	<0.001	12.76 (2.77)	7.68 (3.28)	<0.001
Income, [n(%)]			<0.001			<0.001
<18000	109574 (24.8)	2259 (34.6)		1920 (28.6)	210 (40.5)	
18000-30999	113631 (25.7)	1730 (26.5)		1797 (26.8)	152 (29.3)	
31000-51999	112475 (25.4)	1440 (22.1)		1529 (22.8)	94 (18.1)	
52000-100000	84789 (19.2)	873 (13.4)		1149 (17.1)	46 (8.9)	
>100000	21520 (4.9)	219 (3.4)		317 (4.7)	17 (3.3)	
Smoking status, [n(%)]			<0.001			0.114
Never	243148 (55.0)	2669 (40.9)		2831 (42.2)	203 (39.1)	
Previous	151638 (34.3)	3153 (48.4)		3249 (48.4)	275 (53.0)	
Current	47203 (10.7)	699 (10.7)		632 (9.4)	41 (7.9)	
Drinking status, [n(%)]			0.106			0.141
Yes	406246 (91.9)	6030 (92.5)		6344 (94.5)	482 (92.9)	
No	35743 (8.1)	491 (7.5)		368 (5.5)	37 (7.1)	
Alanine aminotransferase [mean (SD)]	23.38 (14.06)	28.00 (15.92)	<0.001	31.03 (17.44)	29.00 (15.46)	0.01
Aspartate aminotransferase [mean (SD)]	26.09 (10.54)	29.46 (14.13)	<0.001	31.42 (13.66)	31.75 (15.92)	0.604
Alkaline phosphatase [mean (SD)]	83.55 (26.34)	86.55 (28.97)	<0.001	87.46 (27.06)	92.99 (43.88)	<0.001
γ-glutamyltransferase [mean (SD)]	36.69 (40.64)	57.10 (66.46)	<0.001	69.04 (79.94)	73.59 (86.83)	0.214
Polygenic Risk Score [mean (SD)]	−0.05 (0.02)	−0.05 (0.02)	<0.001	–	–	–
eGFR [mean (SD)]	119.78 (16.66)	105.56 (22.63)	<0.001	107.02 (23.13)	98.10 (27.63)	<0.001
Diet score [mean (SD)]	2.68 (1.02)	2.47 (1.05)	<0.001	2.40 (1.08)	2.36 (1.06)	0.419
IMD [mean (SD)]	17.50 (14.02)	19.93 (15.39)	<0.001	18.97 (14.80)	22.58 (17.13)	<0.001
Water intake [(mean (SD)]	2.73 (2.25)	2.78 (2.37)	0.056	3.02 (2.53)	3.33 (2.58)	0.007
Townsend deprivation index [mean (SD)]	−1.27 (3.10)	−0.93 (3.22)	<0.001	−1.04 (3.17)	−0.41 (3.50)	<0.001
Urate [mean (SD)]	305.91 (77.66)	440.16 (88.55)	<0.001	376.36 (101.90)	374.05 (114.83)	0.622
CaCO_3_ concentration [mean (SD)]	130.56 (107.13)	127.69 (104.16)	0.032	124.36 (104.45)	122.15 (104.95)	0.643
Water hardness (WHO), [n(%)]			0.001			0.891
<200 mg/L	298969 (67.6)	4540 (69.6)		4772 (71.1)	371 (71.5)	
≥200 mg/L	143020 (32.4)	1981 (30.4)		1940 (28.9)	148 (28.5)	
Water hardness (USGS), [n(%)]			<0.001			0.722
0–60 mg/L	173713 (39.3)	2535 (38.9)		2700 (40.2)	216 (41.6)	
60–120 mg/L	94606 (21.4)	1521 (23.3)		1575 (23.5)	125 (24.1)	
120–180 mg/L	28285 (6.4)	449 (6.9)		449 (6.7)	29 (5.6)	
> 180 mg/L	145385 (32.9)	2016 (30.9)		1988 (29.6)	149 (28.7)	
Ca concentration [mean (SD)]	51.07 (40.09)	51.20 (39.38)	0.805	50.60 (39.46)	50.91 (38.75)	0.866
Mg concentration [mean (SD)]	4.58 (3.75)	4.60 (3.57)	0.786	4.64 (3.89)	4.89 (4.09)	0.159

Note:SD, Standard Deviation; -, the data on gout recurrence do not include polygenic risk score information.

### Hard water metrics associate with gout incidence

After adjusting age, gender, ethnicity, education levels, Townsend index, IMD, income, BMI, smoking status, drinking status, water intake,urate, ALT, AST, ALP, GGT, PRS and eGFR, articipants exposed to >200 mg/L of hard water had an 13% increased risk of gout (HR: 1.13, 95% CI: 1.07-1.20). Within the USGS classification, the risk increments for groups 60−120 mg/L, 120−180 mg/L and >180 mg/L were 12%, 16%, and 18% respectively, compared to the 0−60 mg/L group. When CaCO_3_, Ca and Mg concentrations were considered as a continuous variable per 50 mg/L increment, the HRs were 1.04 (1.03–1.05) for CaCO_3_, 1.17 (1.13−1.20) for Ca, and 1.99 (1.46–2.71) for Mg. The Q4 group showed a 41% and 19% increased risk for Ca and Mg respectively, compared to Q1 (HR: 1.41 [1.32-1.51] for Ca; HR: 1.19 [1.11–1.27] for Mg). Linear trend tests were significant for WHO classifications and Mg concentration, which is monotonically increasing (all P < 0.05) (Table 2). The RCS curve demonstrated the nonlinear relationship between CaCO_3_ (p for nonlinearity<0.001), Ca (p for nonlinearity = 0.004) and Mg (p for nonlinearity<0.001) concentration and gout incidence (S1-S3 Figs).

**Table 2:** The association between hard water metrics and the risk of gout incidence.

		Model 1		Model 2		
Hard water metrics	Cases/Total	*HRs (95% CIs)*	*P*	*HRs (95% CIs)*	*P*	*P* for trend
WHO (mg/L)						0.0006933
<200	7040/308650	1		1		
≥200	2309/147091	1.06(1.01-1.12)	0.0218	1.13(1.07-1.20)	7.59E-06	
USGS (mg/L)						0.05868
0-60	4104/179162	1		1		
60-120	1995/97824	1.11(1.04-1.18)	0.001854	1.12(1.05-1.19)	0.000695	
120-180	894/29213	1.08(0.98-1.20)	0.117829	1.16(1.05-1.29)	0.003374	
>180	2356/149542	1.11(1.04-1.18)	0.000618	1.18(1.11-1.25)	1.19E-07	
CaCO_3_ concentration (50 mg/L)						
	10120/455741	1.02(1.01-1.03)	0.000338	1.04(1.03-1.05)	1.32E-09	
Ca(50 mg/L)						
	10120/455741	1.11(1.07-1.14)	8.39E-11	1.17(1.13-1.20)	<2e-16	
Q1	3255/143734	1		1		0.458
Q2	2537/113685	1.15(1.08-1.22)	2.91E-05	1.15(1.07-1.22)	4.09E-05	
Q3	1721/87764	1.10(1.02-1.18)	0.0125	1.10(1.02-1.18)	0.013518	
Q4	1836/110558	1.27(1.19-1.36)	1.76E-12	1.41(1.32-1.51)	<2e-16	
Mg (50 mg/L)						
	10120/455741	1.87(1.37-2.54)	7.82E-05	1.99(1.46-2.71)	1.46E-05	
Q1	3241/141391	1		1		0.02526
Q2	2369/102961	1.27(1.19-1.36)	1.65E-12	1.20(1.12-1.28)	1.74E-07	
Q3	1881/106041	1.44(1.34-1.54)	<2e-16	1.52(1.42-1.63)	<2e-16	
Q4	1858/105348	1.21(1.13-1.30)	6.04E-08	1.19(1.11-1.27)	0.000001	

Model 1 was adjusted for age, gender and BMI.

Model 2 was adjusted for age, gender, ethnicity, education levels, Townsend deprivation index, income, BMI, smoking status, drinking status,water intake,urate, ALT, AST, ALP, GGT, PRS and eGFR.

### Hard water metrics associated with gout recurrence

After adjusting covariates (Model 2), the HR (95% CI) of gout recurrence per 50 mg/L increase in Mg concentrations was 2.97 (1.11–7.97). The Q4 group showed a 37% increased risk for Mg compared to Q1, the HR was 1.37 (1.08-1.73). We found a non-significant impact of CaCO_3_ and Ca concentrations on gout recurrence risk. Linear trend test show was significant for Mg, which is monotonically increasing too (P for trend = 0.03026) (Table 3). The exposure-response relationships of CaCO_3_, Ca and Mg concentration with both incident and recurrence gout are shown in S4-S6 Figs.

**Table 3:** The association between hard water metrics and the risk of gout recurrence.

		Model 1		Model 2		
Hard water metrics	Cases/Total	*HRs (95% CIs)*	*P*	*HRs (95% CIs)*	*P*	*P* for trend
WHO (mg/L)						0.8513
<200	371/5143	1		1		
≥200	148/2088	1.01(0.84-1.22)	0.90905	1.10(0.90-1.34)	0.367099	
USGS (mg/L)						0.4524
0-60	216/2916	1		1		
60-120	125/1700	1.00(0.80-1.25)	0.98930	1.04(0.83-1.30)	0.715502	
120-180	29/478	0.78(0.53-1.15)	0.21194	0.87(0.59-1.29)	0.491610	
>180	149/2137	0.97(0.78-1.19)	0.74039	1.07(0.86-1.33)	0.559813	
CaCO_3_ concentration (50 mg/L)						0.00072
	519/7231	1.00(0.96-1.04)	0.88142	1.02(0.97-1.06)	0.45566	
Ca(50 mg/L)						
	519/7231	1.02(0.92-1.14)	0.673558	1.07(0.95-1.19)	0.274225	
Q1	158/2174	1		1		0.7089
Q2	106/2013	1.02(0.82-1.28)	0.838170	1.06(0.85-1.33)	0.609970	
Q3	85/1289	0.88(0.68-1.15)	0.34985	1.00(0.77-1.31)	0.998786	
Q4	126/1755	1.03(0.81-1.30)	0.830457	1.09(0.85-1.39)	0.500474	
Mg (50 mg/L)						
	519/7231	2.26(0.84-6.06)	0.106157	2.97(1.11-7.97)	0.030266	
Q1	140/2172	1		1		0.03026
Q2	106/1553	1.11(0.86-1.42)	0.434927	1.15(0.89-1.48)	0.281834	
Q3	132/1784	1.24(0.98-1.58)	0.076763	1.26(0.99-1.62)	0.062197	
Q4	141/1722	1.31(1.04-1.66)	0.022607	1.37(1.08-1.73)	0.009800	

Model 1 was adjusted for age, gender and BMI.

Model 2 was adjusted for age, gender, ethnicity, education levels, Townsend deprivation index, income, BMI, smoking status, drinking status,water intake,urate, ALT, AST, ALP, GGT and eGFR.

### Stratified the analyses

The age stratification results indicated that patients aged ≥65 years had a higher risk of developing gout when exposed to hard water compared to younger patients (WHO classification>200 mg/L: HR 1.18 [1.07–1.29]; USGS classification>180 mg/L: HR 1.21 [1.09–1.34]). Additionally, Mg concentration also had a significant effect (HR 2.13, 95% CI: 1.26–3.58). The gender stratification results show that continuous exposure of Mg, the risk of gout for females (HR = 4.38, 95% CI: 2.38–8.05, P < 0.001) was significantly higher than males (HR = 1.58, 95% CI: 1.10–2.27, P < 0.05). Similar trends existed in WHO classification (HR = 1.18 for females vs HR = 1.11 for males), USGS>180mg/L (HR = 1.25 for females vs HR = 1.15 for males) and continuous exposure to Ca (HR = 1.22 for females vs HR = 1.14 for males). In addition, our analyses revealed a statistically significant association between the USGS classification and gout risk in BMI ≥ 25 kg/m^2^ group (S3-S5 Tables). The stratified analysis results of gout recurrence showed that among participants aged ≥65 years, the risks of Mg in Q2 and Q3 increased by 57% and 69% respectively compared with Q1, and the HRs were 1.57 (1.02–2.41) and 1.69 (1.10–2.58) respectively. Interaction analysis indicated that age altered the relationship between Mg and gout recurrence, with a stronger positive effect among elderly participants (P _interaction_ <*0.05*) (S6-S8 Tables).

### Sensitivity analysis

Three sensitivity analyses were performed to confirm our findings. First, participants with a follow-up time of < 2 y were removed from the analysis. We found the results were robust for gout incidence and the strength of the association for Mg concentration slightly weakened in gout recurrence compared with the main analysis. Second, we excluded participants with follow-up time of < 5 y. Compared to the main outcome, the relationship between the USGS classification of 60–120 mg/L, Ca concentration and the Q2 classification of Mg and the incidence of gout were slightly weakened, while the correlation of the incidence of > 180 mg/L in USGS was slightly enhanced. We found the association between Mg concentration and gout recurrence was attenuated with the main analysis. Finally, after adjusting hypertension and diabetes, the magnitude of the association was slightly weakened for gout incidence. The relationship was strengthened in Mg and gout recurrence in comparison to the main results (S9 and S10 Tables).

### Supplementary analysis

Based on the second round data, the risk of gout incidence was not significant with the domestic water hardness, CaCO_3_ concentration, Ca and Mg concentration (S11 Table). Only in the USGS classification, the HR (95% CI) of 60−120 mg/L was 1.39 (1.03–1.87), which was positively correlated with the incidence of gout. Due to the small sample size in the second round, it may not meet the requirements of statistical power and is similar to the research results of Zhang et al [23].

## Discussion

In this large population-based cohort of adults from the UK Biobank, we assessed the relationship between domestic hard water and gout. We observed that domestic hard water was positively associated with the incidence of gout. Additionally, Mg concentration in water was found to be positively associated with the recurrence of gout. Furthermore, our research results suggest that high risk groups, such as ≥65 years old, women and those with a BMI ≥ 25 kg/m². The distribution of hard water in the UK varies widely, and our study, which links the geographical locations of participants to their water quality, provides new insights into the relationship between hard water and gout.

To the best of our knowledge, there have been no prior studies investigating the relationship between water hardness and gout, our study attempted to fill this gap in the literature. Some existing studies reported a positive association between water hardness and kidney disease [9,10,24–26]. Formerly, Churchill et al [24] had evaluated urolithiasis in 1,000 hospitals in the USA and found a positive association between water hardness and urinary calculus. A study in the UK reported that the incidence of upper urinary tract stones showed a positively correlated with the total hardness of water [10]. Another observational study also found that higher CaCO_3_ may with higher risk of urolithiasis [25]. A cross-sectional study from England showed a significant positive relationship between Mg concentration and the incidence of COM in one type of urinary tract stones [9]. Besides, a study showed that higher Mg levels in the water may be related to chronic kidney disease of unknown etiology (CKDu) [26].

Although the mechanisms potential the association between hard water and developing gout remain unclear, some studies have already provided possible explanations. Elevated Ca concentration can influence glomerular filtration Ca, leading to increased urinary Ca and saturation of Caoxalate, resulting in crystal blockage of renal tubules [27]. Such blockages may cause renal arteriosclerosis and impair uric acid excretion [28]. Besides, Ca can inhibit cellular Ca pumps, leading to Ca overload and promoting the conversion of xanthine dehydrogenase to xanthine oxidase, which can increase uric acid levels [29].

Current study suggests that Mg deficiency is associated with increased CRP and DNA damage leading to the release of purine nucleotides [12,30]. Moreover, Mg can reduce crystal formation [23], which suggests that Mg may play a potential role in improving renal dysfunction and preventing gout. However, most of these studies are based on plasma Mg levels, and the mechanism by which Mg in domestic water affects gout remains unclear. It is worth noting that there is a complex interaction between Ca and Mg in water. An imbalance in the Ca-to-Mg ratio may promote gout by affecting the excretion of uric acid in the kidneys [31,32]. For instance, a high Ca environment may counteract the benefits of Mg. In addition, other water quality factors may also be involved in regulating this process. Our study found a positive correlation between domestic water high in Ca and Mg and gout incidence, as well as between Mg and gout recurrence. But with a relatively small number of recurrent cases (n = 519), resulting in potentially imprecise hazard ratio estimates and wide confidence intervals (1.11–7.97), these findings should be extrapolated cautiously.

### Strengths and limitations

The main advantage of our study lie its UK Biobank prospective cohort design, which features a large sample size and an extended follow-up period. Our study has some limitations which are worth considering. Firstly, given the observational design of our study, residual confounding remains and causality cannot be inferred. Secondly, Ca and Mg concentrations do not proxy in participants’ bodies, potentially leading to biased or inaccurate conclusions. Thirdly, caution must be exercised when interpreting the results because there is a significant difference in the sample sizes of the two waves of surveys. The number of events surveyed in 2013 was relatively small (272/19,862), which limited the statistical power and might lead to less accurate estimates. These limitations are inherent characteristics of the UK Biobank dataset. Finally, our study is based on a specific population within the UK Biobank, primarily European and mostly white, which may limit the generalization of the findings to other populations. To increase the generality, more other racial and ethnic groups are needed to support the link between gout and water metrics.

## Conclusions

In summary, our study indicated that water hardness, along with Ca and Mg concentrations were positively associated with the incidence of gout, and Mg concentration was associated with an increased risk of gout recurrence. These findings provide new insights into the roles of domestic water hardness in gout and further research is needed to explore geographical factors, and additional environmental and genetic influences, to fully elucidate the relationship between water quality and the epidemiology of gout. As well as interventional investigations in different regions to validate our findings so that develop effective interventions for improving water quality to reduce the incidence of gout and the financial burden on patients. This study highlights the potential of public health strategies that regulate water hardness to prevent gout, which can provide additional scientific basis for formulating water hardness standards. Furthermore, these insights can help guide targeted prevention strategies in high-risk areas, assist in reducing the incidence of gout and its related medical costs, and thereby alleviate the long-term economic burden on individuals and the health system.

## Supporting information

S1 Table Classification of domestic hard water metrics. (DOCX)

S2 Table The numbers and percentages of participants with missing covariates. (DOCX)

S3 Table The association between hard water metrics and risk of gout incidence in stratification analyses for age, gender and BMI. (DOCX)

S4 Table The association between CaCO_3_ and Ca concentration and risk of gout incidence in stratification analyses for age, gender and BMI. (DOCX)

S5 Table The association between Mg concentration and risk of gout incidence in stratification analyses for age, gender and BMI. (DOCX)

S6 Table The association between hard water metrics and risk of gout recurrence in stratification analyses for age, gender and BMI. (DOCX)

S7 Table The association between CaCO_3_ and Ca concentration and risk of gout recurrence in stratification analyses for age, gender and BMI. (DOCX)

S8 Table The association between Mg concentration and risk of gout recurrence in stratification analyses for age, gender and BMI. (DOCX)

S9 Table Sensitivity analysis of the association between hard water metrics and gout incidence. (DOCX)

S10 Table Sensitivity analysis of the association between hard water metrics and gout recurrence. (DOCX)

S11 Table Supplementary analysis of hard water metrics and gout incidence based on the second round of data. (DOCX)

S1 Fig Restricted cubic spline models for the relationship between CaCO_3_ concentration and the risk of gout incidence. (TIF)

S2 Fig Restricted cubic spline models for the relationship between Ca concentration and the risk of gout incidence. (TIF)

S3 Fig Restricted cubic spline models for the relationship between Mg concentration and the risk of gout incidence. (TIF)

S4 Fig Restricted cubic spline models for the relationship between CaCO_3_ concentration and the risk of gout recurrence. (TIF)

S5 Fig Restricted cubic spline models for the relationship between Ca concentration and the risk of gout recurrence. (TIF)

S6 Fig Restricted cubic spline models for the relationship between Mg concentration and the risk of gout recurrence. (TIF)
