# Supplementary material for: Associations of domestic hard water metrics with the risk of gout incidence and recurrence
Source: PLoS One. 2025 Jul 14;20(7):e0326052. doi: 10.1371/journal.pone.0326052 (PMC12258571; doi:10.1371/journal.pone.0326052)
Supplement: S1 Table — (DOCX) [file pone.0326052.s001.docx]

**S1** **Table. Classification of domestic hard water metrics.**

| **Hard water metrics** | **Classification method** | **parameter** |
| --- | --- | --- |
| CaCO3 | continuous variable | 50 mg/L |
|  | WHO classification | >200 mg/L and <200 mg/L |
|  | USGS classification | 0-60 mg/L, 60-120 mg/L, 120-180 mg/L and >180 mg/L |
| Ca and Mg | continuous variable | 50 mg/L |
|  | quartile variable | the unit is 50mg/L |
