# Supplementary material for: Associations of domestic hard water metrics with the risk of gout incidence and recurrence
Source: PLoS One. 2025 Jul 14;20(7):e0326052. doi: 10.1371/journal.pone.0326052 (PMC12258571; doi:10.1371/journal.pone.0326052)
Supplement: S2 Table — (DOCX) [file pone.0326052.s002.docx]

**S2** **Table. The numbers and percentages of participants with missing covariates.**

| **Covariate** | **Incident gout** | | **Recurrent gout** | |
| --- | --- | --- | --- | --- |
|  | **N** | **Percentage** | **N** | **Percentage** |
| Polygenic Risk Score | 111263 | 24.81% | - | - |
| qualifications | 86200 | 19.22% | 47 | 0.65% |
| income | 69242 | 15.44% | 90 | 1.24% |
| aspartate aminotransferase | 30858 | 6.88% | 457 | 6.32% |
| urate | 29776 | 6.64% | 428 | 5.92% |
| creatinine | 29457 | 6.57% | 427 | 5.91% |
| gamma glutamyltransferase | 29470 | 6.57% | 433 | 5.99% |
| alanine aminotransferase | 29413 | 6.56% | 434 | 5.99% |
| cystatin c | 29290 | 6.53% | 425 | 5.88% |
| alkaline phosphatase | 29251 | 6.52% | 422 | 5.84% |
| IMD | 11213 | 2.50% | 182 | 2.52% |
| salad raw vegetable intake | 6871 | 1.53% | 7 | 0.10% |
| cooked vegetable intake | 6608 | 1.47% | 7 | 0.10% |
| water intake | 4170 | 0.93% | 7 | 0.10% |
| lambmutton intake | 3926 | 0.88% | 7 | 0.10% |
| pork intake | 3749 | 0.84% | 7 | 0.10% |
| oily fish intake | 3506 | 0.78% | 7 | 0.10% |
| nonoily fish intake | 3196 | 0.71% | 7 | 0.10% |
| beef intake | 2829 | 0.63% | 7 | 0.10% |
| fresh fruit intake | 2731 | 0.61% | 7 | 0.10% |
| bmi | 2657 | 0.59% | 41 | 0.57% |
| smoking status | 2493 | 0.56% | 7 | 0.10% |
| ethnic | 2340 | 0.52% | 7 | 0.10% |
| processed meat intake | 1853 | 0.41% | 7 | 0.10% |
| drinking status | 1221 | 0.27% | 7 | 0.10% |
| townsend deprivation index at recruitment | 487 | 0.11% | 8 | 0.11% |
