# Supplementary material for: Associations of domestic hard water metrics with the risk of gout incidence and recurrence
Source: PLoS One. 2025 Jul 14;20(7):e0326052. doi: 10.1371/journal.pone.0326052 (PMC12258571; doi:10.1371/journal.pone.0326052)
Supplement: S3 Table — (DOCX) [file pone.0326052.s003.docx]

**S3** **Table. The association between hard water and risk of gout incidence in stratification analyses for age, gender and BMI.**

| **Subgroup** | **WHO (mg/L)** | | **P _Interaction_** | **USGS (mg/L)** | | | | **P _Interaction_** |
| --- | --- | --- | --- | --- | --- | --- | --- | --- |
|  | **＜200** | **≥200** |  | **0-60** | **60-120** | **120-180** | **＞180** |  |
| **Age group ^a^** |  |  |  |  |  |  |  |  |
| ＜65 | 1.00 | 1.10(1.02-1.17)** | 0.08 | 1.00 | 1.15(1.06-1.24)*** | 1.16(1.02-1.31) | 1.14(1.05-1.27)** | 0.02386* |
| ≥65 | 1.00 | 1.18(1.07-1.29)*** |  | 1.00 | 1.03(0.92-1.15) | 1.15(0.97-1.37) | 1.21(1.09-1.34)*** |  |
| **Gender group ^b^** |  |  |  |  |  |  |  |  |
| Male | 1.00 | 1.11(1.05-1.19)*** | 0.8645 | 1.00 | 1.10(1.02-1.18)* | 1.17(1.04-1.31) | 1.15(1.07-1.23)*** | 0.7706 |
| Female | 1.00 | 1.18(1.05-1.32)** |  | 1.00 | 1.18(1.03-1.35)* | 1.10(0.88-1.37) | 1.25(1.10-1.42)*** |  |
| **BMI group ^c^** |  |  |  |  |  |  |  |  |
| ＜25 kg/m^2^ | 1.00 | 1.17(0.99-1.37) | 0.06847 | 1.00 | 1.03(0.85-1.26) | 0.80(0.57-1.13) | 1.03(0.87-1.23) | 0.1372 |
| ≥25 kg/m^2^ | 1.00 | 1.10(1.04-1.16)** |  | 1.00 | 1.11(1.03-1.18)** | 1.19(1.07-1.32)** | 1.16(1.08-1.23)*** |  |

^a^ was adjusted for gender, ethnicity, education levels, Townsend deprivation index, income, BMI, smoking status, drinking status, water intake, urate, ALT, AST, ALP, GGT, PRS and eGFR. ^b^ further adjusted for age (instead of gender), with other covariates matching the a model. ^c^ was adjusted for age and gender (instead of BMI), with other covariates consistent. ***P＜0.001, **P < 0.01, *P<0.05.
