# Supplementary material for: Associations of domestic hard water metrics with the risk of gout incidence and recurrence
Source: PLoS One. 2025 Jul 14;20(7):e0326052. doi: 10.1371/journal.pone.0326052 (PMC12258571; doi:10.1371/journal.pone.0326052)
Supplement: S4 Table — (DOCX) [file pone.0326052.s004.docx]

**S4** **Table. The association between CaCO3 and Ca concentration and risk of gout incidence in stratification analyses for age, gender and BMI.**

| **Subgroup** | **CaCO3 concentration (50 mg/L)** | **P _Interaction_** | **Ca(50 mg/L)** | **P _Interaction_** | **Ca(50 mg/L)** | | | | **P _Interaction_** |
| --- | --- | --- | --- | --- | --- | --- | --- | --- | --- |
|  |  |  |  |  | **Q1** | **Q2** | **Q3** | **Q4** |  |
| **Age group ^a^** |  |  |  |  |  |  |  |  |  |
| ＜65 | 1.03(1.01-1.05)*** | 0.1313 | 1.16(1.12-1.21)*** | 0.6378 | 1.00 | 1.16(1.07-1.26)*** | 1.06(0.97-1.17) | 1.40(1.29-1.53)*** | 0.2534 |
| ≥65 | 1.04(1.02-1.07)*** |  | 1.16(1.10-1.22)*** |  | 1.00 | 1.09(0.97-1.22) | 1.14(1.01-1.28)* | 1.38(1.22-1.55)*** |  |
| **Gender group ^b^** |  |  |  |  |  |  |  |  |  |
| Male | 1.03(1.02-1.05)*** | 0.8016 | 1.14(1.10-1.19)*** | 0.4812 | 1.00 | 1.10(1.02-1.18)* | 1.03(0.95-1.12) | 1.37(1.27-1.48)*** | 0.136 |
| Female | 1.04(1.02-1.07)*** |  | 1.22(1.14-1.30)*** |  | 1.00 | 1.31(1.14-1.51)*** | 1.34(1.15-1.55)*** | 1.48(1.28-1.72)*** |  |
| **BMI group ^c^** |  |  |  |  |  |  |  |  |  |
| ＜25 kg/m^2^ | 1.02(0.98-1.06) | 0.2728 | 1.18(1.07-1.29)*** | 0.007052 ** | 1.00 | 1.35(1.11-1.64)** | 0.93(0.74-1.16) | 1.57(1.28-1.92)*** | 1.257e-07 *** |
| ≥25 kg/m^2^ | 1.03(1.02-1.05)*** |  | 1.14(1.10-1.18)*** |  | 1.00 | 1.09(1.02-1.17)* | 1.07(0.99-1.16) | 1.32(1.23-1.42)*** |  |

^a^ was adjusted for gender, ethnicity, education levels, Townsend deprivation index, income, BMI, smoking status, drinking status, water intake, urate, ALT, AST, ALP, GGT, PRS and eGFR. ^b^ further adjusted for age (instead of gender), with other covariates matching the a model. ^c^ was adjusted for age and gender (instead of BMI), with other covariates consistent. ***P＜0.001, **P < 0.01, *P<0.05.
