# Supplementary material for: Associations of domestic hard water metrics with the risk of gout incidence and recurrence
Source: PLoS One. 2025 Jul 14;20(7):e0326052. doi: 10.1371/journal.pone.0326052 (PMC12258571; doi:10.1371/journal.pone.0326052)
Supplement: S5 Table — (DOCX) [file pone.0326052.s005.docx]

**S5** **Table. The association between Mg concentration and risk of gout incidence in stratification analyses for age, gender and BMI.**

| **Subgroup** | **Mg(50 mg/L)** | **P _Interaction_** | **Mg(50 mg/L)** | | | | **P _Interaction_** |
| --- | --- | --- | --- | --- | --- | --- | --- |
|  |  |  | **Q1** | **Q2** | **Q3** | **Q4** |  |
| **Age group ^a^** |  |  |  |  |  |  |  |
| ＜65 | 1.85(1.25-2.72)** | 0.8388 | 1.00 | 1.13(1.04-1.23)** | 1.50(1.37-1.63)*** | 1.19(1.09-1.30)*** | 0.04345* |
| ≥65 | 2.13(1.26-3.58)** |  | 1.00 | 1.38(1.23-1.55)*** | 1.55(1.38-1.75)*** | 1.18(1.05-1.33)** |  |
| **Gender group ^b^** |  |  |  |  |  |  |  |
| Male | 1.58(1.10-2.27)* | 0.06344 | 1.00 | 1.22(1.13-1.32)*** | 1.48(1.37-1.60)*** | 1.12(1.04-1.21)** | 0.04199* |
| Female | 4.38(2.38-8.05)*** |  | 1.00 | 1.24(1.07-1.44)** | 1.63(1.41-1.89)*** | 1.48(1.28-1.70)*** |  |
| **BMI group ^c^** |  |  |  |  |  |  |  |
| ＜25 kg/m^2^ | 1.38(0.51-3.72) | 0.7932 | 1.00 | 1.01(0.81-1.24) | 1.71(1.40-2.09)*** | 1.14(0.93-1.40) | 7.905e-12 *** |
| ≥25 kg/m^2^ | 1.93(1.39-2.69)*** |  | 1.00 | 1.31(1.22-1.41)*** | 1.48(1.37-1.59)*** | 1.20(1.12-1.30)*** |  |

^a^ was adjusted for gender, ethnicity, education levels, Townsend deprivation index, income, BMI, smoking status, drinking status, water intake, urate, ALT, AST, ALP, GGT, PRS and eGFR. ^b^ further adjusted for age (instead of gender), with other covariates matching the a model. ^c^ was adjusted for age and gender (instead of BMI), with other covariates consistent. ***P＜0.001, **P < 0.01, *P<0.05.
