# Supplementary material for: Associations of domestic hard water metrics with the risk of gout incidence and recurrence
Source: PLoS One. 2025 Jul 14;20(7):e0326052. doi: 10.1371/journal.pone.0326052 (PMC12258571; doi:10.1371/journal.pone.0326052)
Supplement: S6 Table — (DOCX) [file pone.0326052.s006.docx]

**S6** **Table. The association between hard water and risk of gout recurrence in stratification analyses for age, gender and BMI.**

| **Subgroup** | **WHO (mg/L)** | | **P _Interaction_** | **USGS (mg/L)** | | | | **P _Interaction_** |
| --- | --- | --- | --- | --- | --- | --- | --- | --- |
|  | **＜200** | **≥200** |  | **0-60** | **60-120** | **120-180** | **＞180** |  |
| **Age group ^a^** |  |  |  |  |  |  |  |  |
| ＜65 | 1.00 | 1.19(0.93-1.52) | 0.44 | 1.00 | 1.08(0.82-1.42) | 1.11(0.71-1.74) | 1.21(0.92-1.58) | 0.1885 |
| ≥65 | 1.00 | 0.96(0.67-1.36) |  | 1.00 | 0.93(0.64-1.36) | 0.45(0.20-1.04) | 0.84(0.58-1.23) |  |
| **Gender group ^b^** |  |  |  |  |  |  |  |  |
| Male | 1.00 | 1.15(0.94-1.42) | 0.36 | 1.00 | 1.07(0.85-1.34) | 0.92(0.62-1.38) | 1.13(0.90-1.42) | 0.6838 |
| Female | 1.00 | 0.61(0.28-1.36) |  | 1.00 | 0.73(0.33-1.64) | 0.45(0.10-2.01) | 0.50(0.22-1.18) |  |
| **BMI group ^c^** |  |  |  |  |  |  |  |  |
| ＜25 kg/m^2^ | 1.00 | 0.78(0.36-1.65) | 0.27441 | 1.00 | 0.49(0.19-1.24) | 0.97(0.27-3.45) | 0.61(0.28-1.35) | 0.2099 |
| ≥25 kg/m^2^ | 1.00 | 1.12(0.91-1.39) |  | 1.00 | 1.10(0.87-1.39) | 0.89(0.59-1.34) | 1.12(0.89-1.41) |  |

^a^ was adjusted for gender, ethnicity, education levels, Townsend deprivation index, income, BMI, smoking status, drinking status, water intake, urate, ALT, AST, ALP, GGT and eGFR. ^b^ further adjusted for age (instead of gender), with other covariates matching the a model. ^c^ was adjusted for age and gender (instead of BMI), with other covariates consistent. ***P＜0.001, **P < 0.01, *P<0.05.
