# Supplementary material for: Associations of domestic hard water metrics with the risk of gout incidence and recurrence
Source: PLoS One. 2025 Jul 14;20(7):e0326052. doi: 10.1371/journal.pone.0326052 (PMC12258571; doi:10.1371/journal.pone.0326052)
Supplement: S7 Table — (DOCX) [file pone.0326052.s007.docx]

**S7** **Table. The association between CaCO3 and Ca concentration and risk of gout recurrence in stratification analyses for age, gender and BMI.**

| **Subgroup** | **CaCO3 concentration (50 mg/L)** | **P _Interaction_** | **Ca(50 mg/L)** | **P _Interaction_** | **Ca(50 mg/L)** | | | | **P _Interaction_** |
| --- | --- | --- | --- | --- | --- | --- | --- | --- | --- |
|  |  |  |  |  | **Q1** | **Q2** | **Q3** | **Q4** |  |
| **Age group ^a^** |  |  |  |  |  |  |  |  |  |
| ＜65 | 1.03(0.98-1.09) | 0.2986 | 1.07(0.93-1.23) | 0.7622 | 1.00 | 1.14(0.86-1.51) | 1.22(0.89-1.68) | 1.05(0.77-1.43) | 0.05446 |
| ≥65 | 0.98(0.90-1.05) |  | 1.06(0.87-1.29) |  | 1.00 | 0.91(0.62-1.34) | 0.65(0.39-1.08) | 1.12(0.75-1.67) |  |
| **Gender group ^b^** |  |  |  |  |  |  |  |  |  |
| Male | 1.03(0.98-1.08) | 0.2678 | 1.05(0.93-1.18) | 0.1548 | 1.00 | 1.07(0.85-1.35) | 1.03(0.78-1.36) | 1.03(0.80-1.33) | 0.1983 |
| Female | 0.88(0.74-1.04) |  | 1.32(0.87-2.01) |  | 1.00 | 0.82(0.33-2.05) | 0.65(0.22-1.94) | 1.79(0.78-4.10) |  |
| **BMI group ^c^** |  |  |  |  |  |  |  |  |  |
| ＜25 kg/m^2^ | 0.92(0.78-1.08) | 0.1211 | 0.82(0.53-1.26) | 0.1303 | 1.00 | 0.72(0.32-1.61) | 0.99(0.41-2.42) | 0.49(0.19-1.31) | 0.31 |
| ≥25 kg/m^2^ | 1.02(0.98-1.07) |  | 1.09(0.97-1.23) |  | 1.00 | 1.09(0.86-1.53) | 1.02(0.77-1.35) | 1.15(0.89-1.48) |  |

^a^ was adjusted for gender, ethnicity, education levels, Townsend deprivation index, income, BMI, smoking status, drinking status, water intake, urate, ALT, AST, ALP, GGT and eGFR. ^b^ further adjusted for age (instead of gender), with other covariates matching the a model. ^c^ was adjusted for age and gender (instead of BMI), with other covariates consistent. ***P＜0.001, **P < 0.01, *P<0.05.
