# Supplementary material for: Associations of domestic hard water metrics with the risk of gout incidence and recurrence
Source: PLoS One. 2025 Jul 14;20(7):e0326052. doi: 10.1371/journal.pone.0326052 (PMC12258571; doi:10.1371/journal.pone.0326052)
Supplement: S8 Table — (DOCX) [file pone.0326052.s008.docx]

**S8** **Table. The association between Mg concentration and risk of gout recurrence in stratification analyses for age, gender and BMI.**

| **Subgroup** | **Mg(50 mg/L)** | **P _Interaction_** | **Mg(50 mg/L)** | | | | **P _Interaction_** |
| --- | --- | --- | --- | --- | --- | --- | --- |
|  |  |  | **Q1** | **Q2** | **Q3** | **Q4** |  |
| **Age group ^a^** |  |  |  |  |  |  |  |
| ＜65 | 6.04(1.97-18.48)** | 0.04419 * | 1.00 | 0.95(0.69-1.31) | 1.07(0.78-1.45) | 1.48(1.12-1.96)** | 0.003067 ** |
| ≥65 | 0.60(0.07-4.76) |  | 1.00 | 1.57(1.02-2.41)* | 1.69(1.10-2.58)* | 1.12(0.72-1.76) |  |
| **Gender group ^b^** |  |  |  |  |  |  |  |
| Male | 3.08(1.10-8.60)* | 0.9969 | 1.00 | 1.12(0.86-1.46) | 1.17(0.91-1.52) | 1.36(1.07-1.74)* | 0.1447 |
| Female | 1.97(0.03-139.18) |  | 1.00 | 1.59(0.60-4.24) | 2.54(1.06-6.11)* | 1.26(0.46-3.48) |  |
| **BMI group ^c^** |  |  |  |  |  |  |  |
| ＜25 kg/m^2^ | 0.51(0.10-60.05) | 0.3682 | 1.00 | 0.81(0.34-1.97) | 0.56(0.21-1.52) | 1.13(0.51-2.48) | 0.3437 |
| ≥25 kg/m^2^ | 3.22(1.18-8.82)* |  | 1.00 | 1.17(0.90-1.53) | 1.33(1.03-1.71)* | 1.38(1.07-1.76)* |  |

^a^ was adjusted for gender, ethnicity, education levels, Townsend deprivation index, income, BMI, smoking status, drinking status, water intake, urate, ALT, AST, ALP, GGT and eGFR. ^b^ further adjusted for age (instead of gender), with other covariates matching the a model. ^c^ was adjusted for age and gender (instead of BMI), with other covariates consistent. ***P＜0.001, **P < 0.01, *P<0.05.
