# Supplementary material for: Associations of domestic hard water metrics with the risk of gout incidence and recurrence
Source: PLoS One. 2025 Jul 14;20(7):e0326052. doi: 10.1371/journal.pone.0326052 (PMC12258571; doi:10.1371/journal.pone.0326052)
Supplement: S9 Table — (DOCX) [file pone.0326052.s009.docx]

**S9** **Table. Sensitivity analysis of the association between hard water metrics and gout incidence.**

| **Hard water metrics** | | **Model a** | | **Model b** | | **Model c** | |
| --- | --- | --- | --- | --- | --- | --- | --- |
|  |  | ***HRs* (*95% CIs*)** | ***P*** | ***HRs* (*95% CIs*)** | ***P*** | ***HRs (95% CIs)*** | ***P*** |
| WHO (mg/L) | |  |  |  |  |  |  |
| ＜200 | | 1 |  | 1 |  | 1 |  |
| ＞200 | | 1.13(1.07-1.20) | 1.48E-05 | 1.14(1.07-1.20) | 1.83E-05 | 1.12(1.06-1.18) | 6.22E-05 |
| USGS (mg/L) | |  |  |  |  |  |  |
| 0-60 | | 1 |  | 1 |  | 1 |  |
| 60-120 | | 1.13(1.06-1.21) | 2.14E-04 | 1.11(1.03-1.18) | 0.003665 | 1.10(1.03-1.18) | 0.002929 |
| 120-180 | | 1.16(1.04-1.28) | 0.005149 | 1.16(1.04-1.29) | 5.91E-03 | 1.16(1.04-1.28) | 0.004970 |
| ＞180 | | 1.20(1.13-1.28) | 3.96E-09 | 1.20(1.12-1.28) | 2.42E-08 | 1.15(1.09-1.23) | 3.44E-06 |
| CaCO_3_ concentration(50 mg/L) | |  |  |  |  |  |  |
|  | | 1.04(1.02-1.05) | 5.08E-09 | 1.04(1.02-1.05) | 2.14E-08 | 1.03(1.02-1.05) | 3.42E-08 |
| Ca(50 mg/L) | |  |  |  |  |  |  |
|  | | 1.16(1.13-1.20) | < 2e-16 | 1.16(1.12-1.20) | < 2e-16 | 1.16(1.12-1.19) | < 2e-16 |
| Q1 | | 1 |  | 1 |  | 1 |  |
| Q2 | | 1.15(1.07-1.22) | 5.58E-05 | 1.13(1.06-1.21) | 0.000436 | 1.14(1.06-1.21) | 1.44E-04 |
| Q3 | | 1.09(1.02-1.18) | 0.018275 | 1.09(1.01-1.18) | 0.023811 | 1.09(1.01-1.17) | 0.022820 |
| Q4 | | 1.41(1.31-1.51) | < 2e-16 | 1.39(1.30-1.50) | < 2e-16 | 1.39(1.30-1.49) | < 2e-16 |
| Mg (50 mg/L) | |  |  |  |  |  |  |
|  | | 1.99(1.45-2.72) | 1.91E-05 | 2.00(1.44-2.77) | 3.80E-05 | 1.98(1.45-2.71) | 1.77E-05 |
| Q1 | | 1 |  | 1 |  | 1 |  |
| Q2 | | 1.20(1.12-1.28) | 3.31E-07 | 1.20(1.12-1.29) | 4.12E-07 | 1.20(1.12-1.28) | 2.75E-07 |
| Q3 | | 1.51(1.41-1.62) | < 2e-16 | 1.49(1.38-1.61) | < 2e-16 | 1.51(1.41-1.62) | < 2e-16 |
| Q4 | | 1.18(1.10-1.27) | 2.64E-06 | 1.19(1.10-1.28) | 3.48E-06 | 1.18(1.10-1.27) | 2.47E-06 |
| Model a: Participants with a follow-up of less than 2 years were excluded | | | | | | | |
| Model b: Participants with a follow-up of less than 5 years were excluded | | | | | | | |
| Model c: Model 2 is based on the addition of hypertension and diabetes | | | | | | | |
