# Supplementary material for: Associations of domestic hard water metrics with the risk of gout incidence and recurrence
Source: PLoS One. 2025 Jul 14;20(7):e0326052. doi: 10.1371/journal.pone.0326052 (PMC12258571; doi:10.1371/journal.pone.0326052)
Supplement: S10 Table — (DOCX) [file pone.0326052.s010.docx]

**S10** **Table. Sensitivity analysis of the association between hard water metrics and gout recurrence.**

| **Hard water metrics** | | **Model a** | | **Model b** | | **Model c** | |
| --- | --- | --- | --- | --- | --- | --- | --- |
|  |  | ***HRs* (*95% CIs*)** | ***P*** | ***HRs* (*95% CIs*)** | ***P*** | ***HRs (95% CIs)*** | ***P*** |
| WHO (mg/L) | |  |  |  |  |  |  |
| ＜200 | | 1 |  | 1 |  | 1 |  |
| ＞200 | | 1.06(0.86-1.30) | 0.592984 | 0.97(0.76-1.23) | 0.80094 | 1.10(0.90-1.35) | 0.354340 |
| USGS (mg/L) | |  |  |  |  |  |  |
| 0-60 | | 1 |  | 1 |  | 1 |  |
| 60-120 | | 1.06(0.85-1.32) | 0.622982 | 1.01(0.78-1.29) | 0.96683 | 1.04(0.84-1.30) | 0.714035 |
| 120-180 | | 0.89(0.60-1.31) | 0.554821 | 0.98(0.64-1.48) | 0.90770 | 0.92(0.62-1.36) | 0.675991 |
| ＞180 | | 1.04(0.83-1.30) | 0.754577 | 0.95(0.73-1.22) | 0.66822 | 1.07(0.86-1.34) | 0.525381 |
| CaCO_3_ concentration(50 mg/L) | |  |  |  |  |  |  |
|  | | 1.01(0.96-1.05) | 0.733450 | 0.99(0.94-1.04) | 0.66598 | 1.02(0.97-1.06) | 0.414465 |
| Ca(50 mg/L) | |  |  |  |  |  |  |
|  | | 1.06(0.94-1.19) | 0.326580 | 1.03(0.90-1.18) | 0.64967 | 1.07(0.95-1.20) | 0.25202 |
| Q1 | | 1 |  | 1 |  | 1 |  |
| Q2 | | 1.07(0.85-1.34) | 0.585651 | 1.03(0.80-1.34) | 0.80704 | 1.05(0.84-1.32) | 0.653524 |
| Q3 | | 1.01(0.77-1.32) | 0.953815 | 1.03(0.77-1.39) | 0.83120 | 1.02(0.78-1.34) | 0.856963 |
| Q4 | | 1.07(0.83-1.37) | 0.600397 | 0.98(0.74-1.31) | 0.90542 | 1.09(0.86-1.40) | 0.473096 |
| Mg (50 mg/L) | |  |  |  |  |  |  |
|  | | 2.80(1.02-7.63) | 0.04486 | 3.56(1.20-10.56) | 0.02198 | 3.21(1.19-8.63) | 0.020863 |
| Q1 | | 1 |  | 1 |  | 1 |  |
| Q2 | | 1.14(0.88-1.48) | 0.315920 | 0.96(0.71-1.30) | 0.80383 | 1.14(0.89-1.48) | 0.299484 |
| Q3 | | 1.23(0.96-1.58) | 0.107205 | 1.11(0.83-1.47) | 0.47674 | 1.27(0.99-1.62) | 0.058772 |
| Q4 | | 1.38(1.09-1.75) | 0.007764 | 1.38(1.06-1.79) | 0.01701 | 1.37(1.09-1.74) | 0.008382 |
| Model a: Participants with a follow-up of less than 2 years were excluded | | | | | | | |
| Model b: Participants with a follow-up of less than 5 years were excluded | | | | | | | |
| Model c: Model 2 is based on the addition of hypertension and diabetes | | | | | | | |
