# Supplementary material for: Associations of domestic hard water metrics with the risk of gout incidence and recurrence
Source: PLoS One. 2025 Jul 14;20(7):e0326052. doi: 10.1371/journal.pone.0326052 (PMC12258571; doi:10.1371/journal.pone.0326052)
Supplement: S11 Table — (DOCX) [file pone.0326052.s011.docx]

**S11 Table Supplementary analysis of hard water metrics and gout incidence based on the second round of data.**

| **Hard water metrics** | **Cases/Total** | **Model 1** | | **Model 2** | |
| --- | --- | --- | --- | --- | --- |
|  |  | ***HRs (95% CIs)*** | ***P*** | ***HRs (95% CIs)*** | ***P*** |
| WHO (mg/L) |  |  |  |  |  |
| ＜200 | 262/18995 | 1 |  | 1 |  |
| ≥200 | 10/867 | 0.95(0.50-1.79) | 0.873 | 1.02(0.54-1.92) | 0.957919 |
| USGS (mg/L) |  |  |  |  |  |
| 0-60 | 183/13697 | 1 |  | 1 |  |
| 60-120 | 60/3761 | 1.32(0.98-1.76) | 0.064 | 1.39(1.03-1.87) | 0.030218 |
| 120-180 | 18/1384 | 1.08(0.67-1.76) | 0.745 | 1.03(0.63-1.69) | 0.896740 |
| ＞180 | 11/1020 | 0.96(0.52-1.77) | 0.901 | 1.04(0.56-1.93) | 0.892316 |
| CaCO3 concentration (50 mg/L) |  |  |  |  |  |
|  | 272/19862 | 1.00(0.99-1.00) | 0.587 | 1.04(0.94-1.16) | 0.437654 |
| Ca(50 mg/L) |  |  |  |  |  |
|  | 272/19862 | 1.08(0.77-1.51) | 0.655 | 1.09(0.78-1.52) | 0.629483 |
| Q1 | 73/5502 | 1 |  | 1 |  |
| Q2 | 70/5090 | 0.92(0.66-1.28) | 0.615 | 0.97(0.69-1.35) | 0.843476 |
| Q3 | 60/4415 | 1.03(0.73-1.45) | 0.876 | 1.07(0.76-1.52) | 0.695832 |
| Q4 | 69/4855 | 1.10(0.79-1.53) | 0.566 | 1.15(0.82-1.61) | 0.413430 |
| Mg (50 mg/L) |  |  |  |  |  |
|  | 272/19862 | 1.01(0.19-5.34) | 0.991 | 1.33(0.24-7.30) | 0.742709 |
| Q1 | 71/5472 | 1 |  | 1 |  |
| Q2 | 102/7440 | 1.20(0.88-1.62) | 0.253 | 1.13(0.83-1.53) | 0.452643 |
| Q3 | 30/2010 | 1.36(0.89-2.09) | 0.157 | 1.25(0.81-1.92) | 0.318150 |
| Q4 | 69/4940 | 1.26(0.90-1.77) | 0.174 | 1.29(0.92-1.80) | 0.144815 |

Model 1 was adjusted for age, gender and BMI.

Model 2 was adjusted for age, gender, ethnicity, education levels, Townsend deprivation index, income, BMI, smoking status, drinking status, water intake, urate, ALT, AST, ALP, GGT, PRS and eGFR.
