# Supplementary figures and images for: Associations of domestic hard water metrics with the risk of gout incidence and recurrence
Source: PLoS One. 2025 Jul 14;20(7):e0326052. doi: 10.1371/journal.pone.0326052 (PMC12258571; doi:10.1371/journal.pone.0326052)

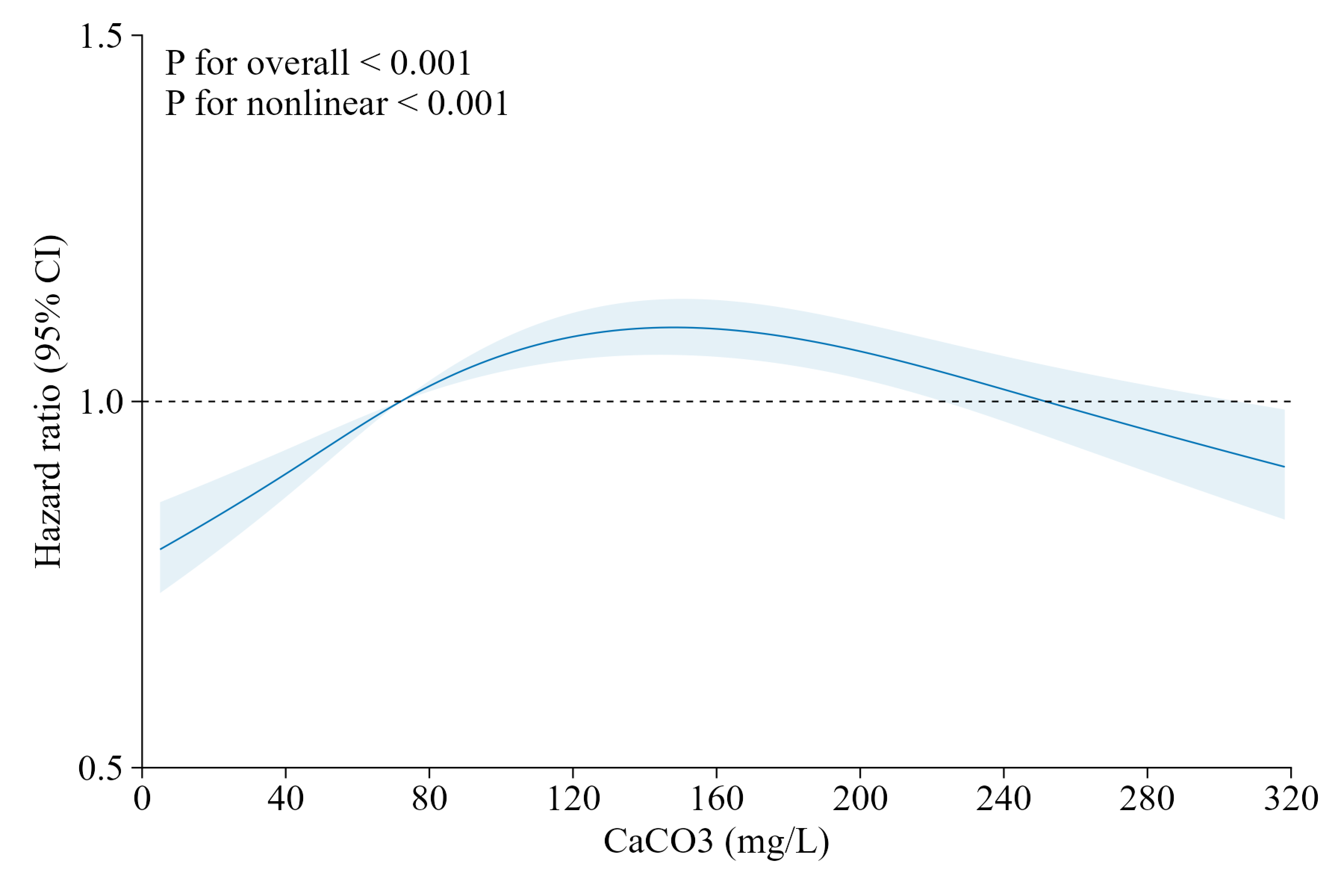

Supplement: S1 Fig — (TIF) [file pone.0326052.s012.tif]

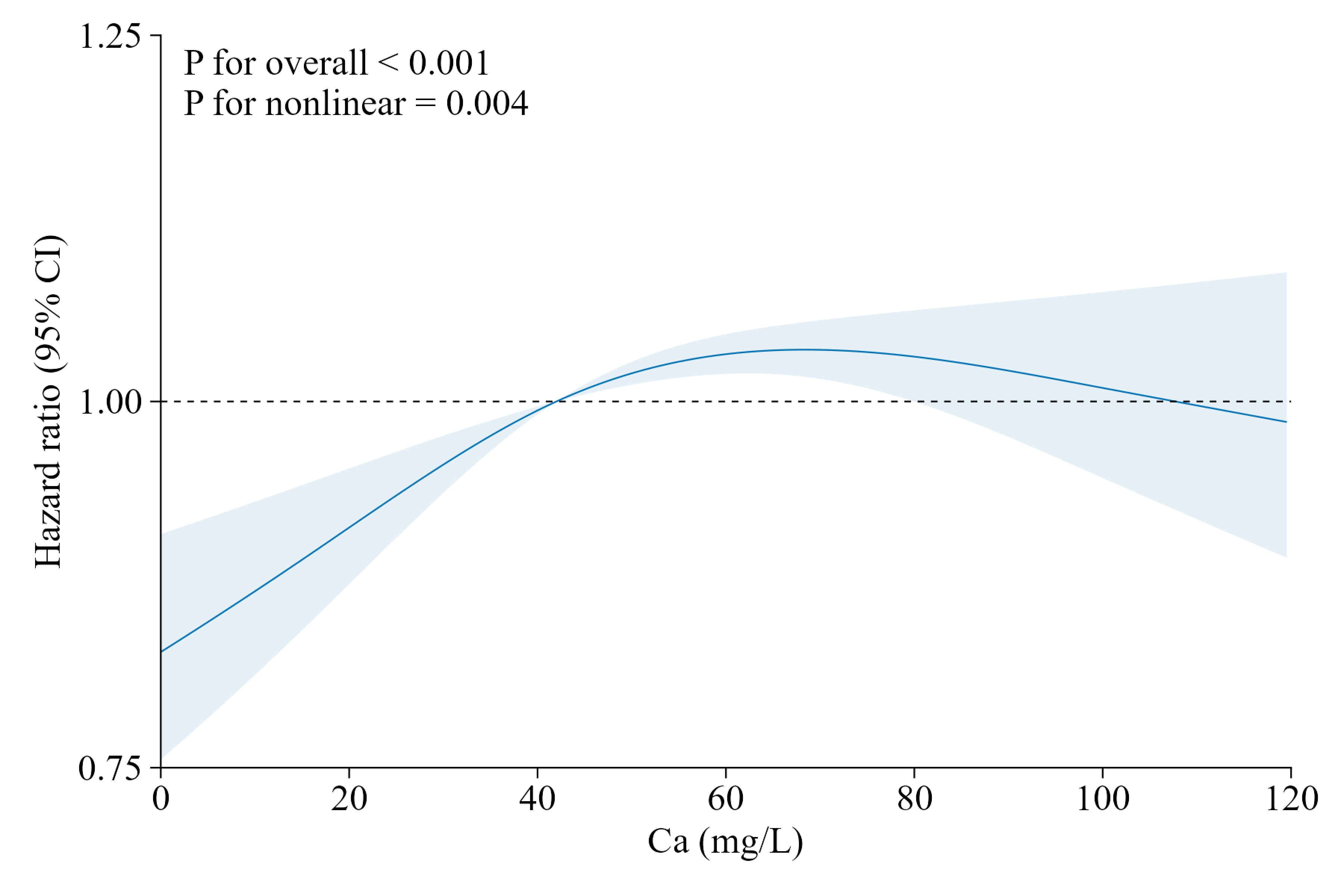

Supplement: S2 Fig — (TIF) [file pone.0326052.s013.tif]

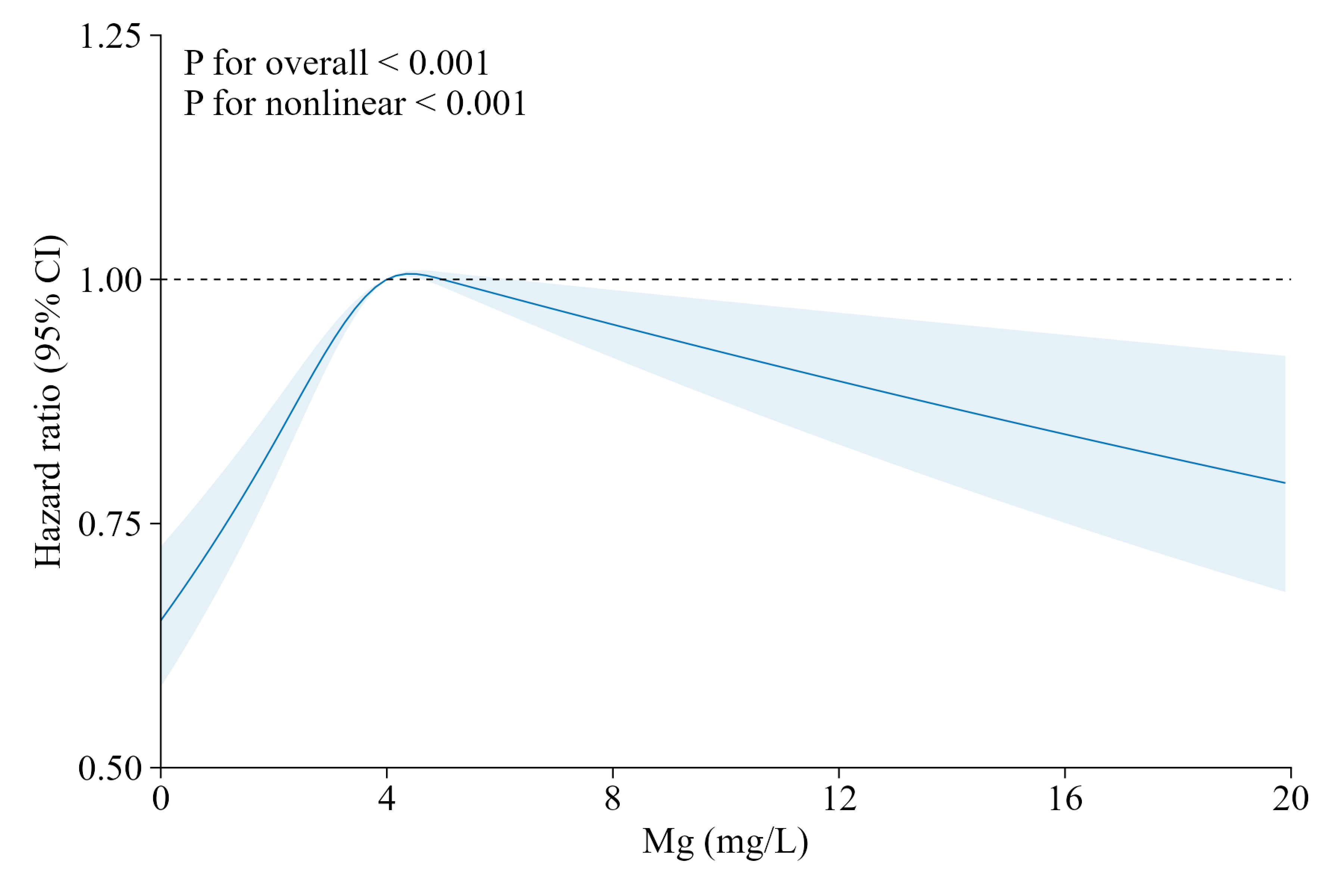

Supplement: S3 Fig — (TIF) [file pone.0326052.s014.tif]

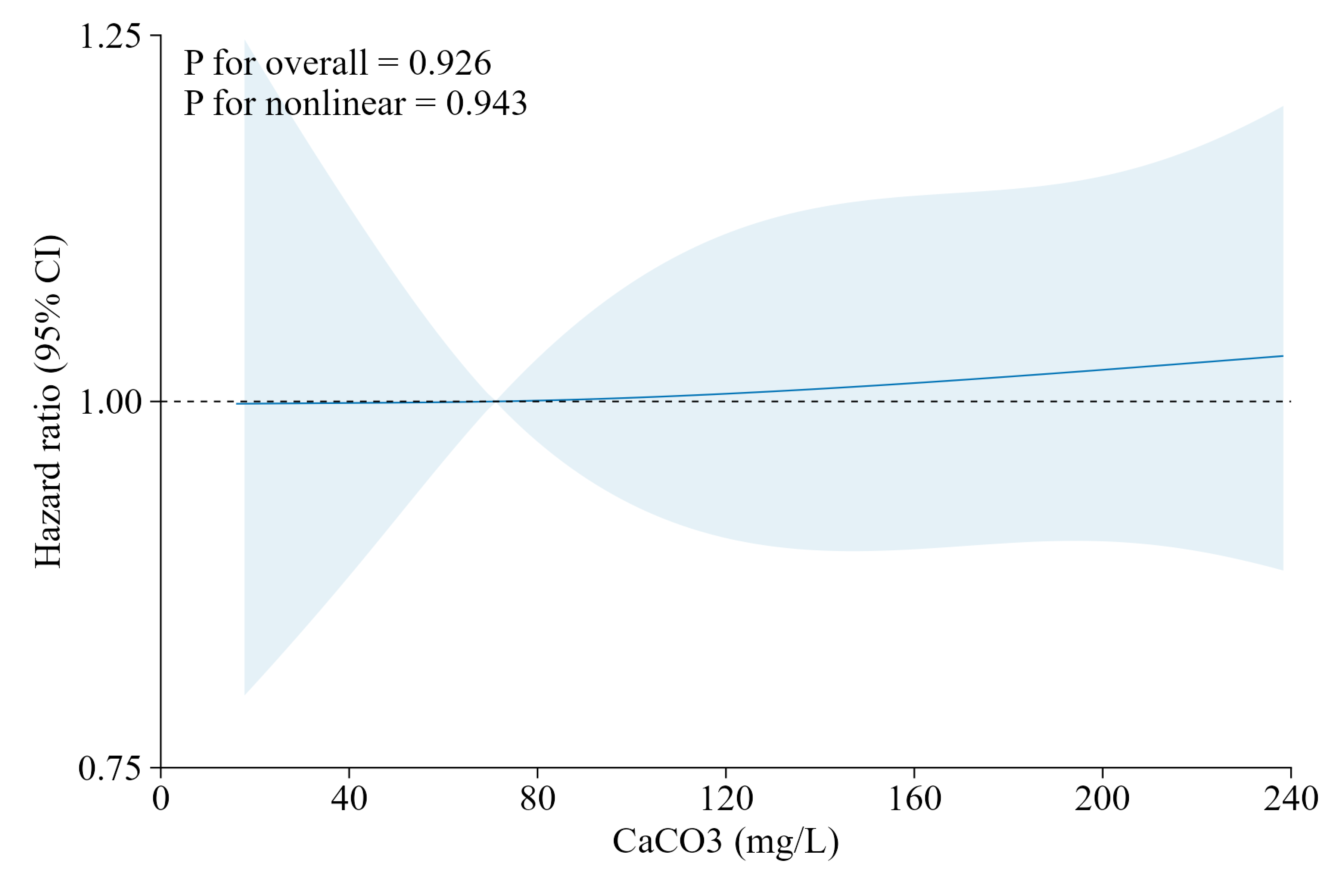

Supplement: S4 Fig — (TIF) [file pone.0326052.s015.tif]

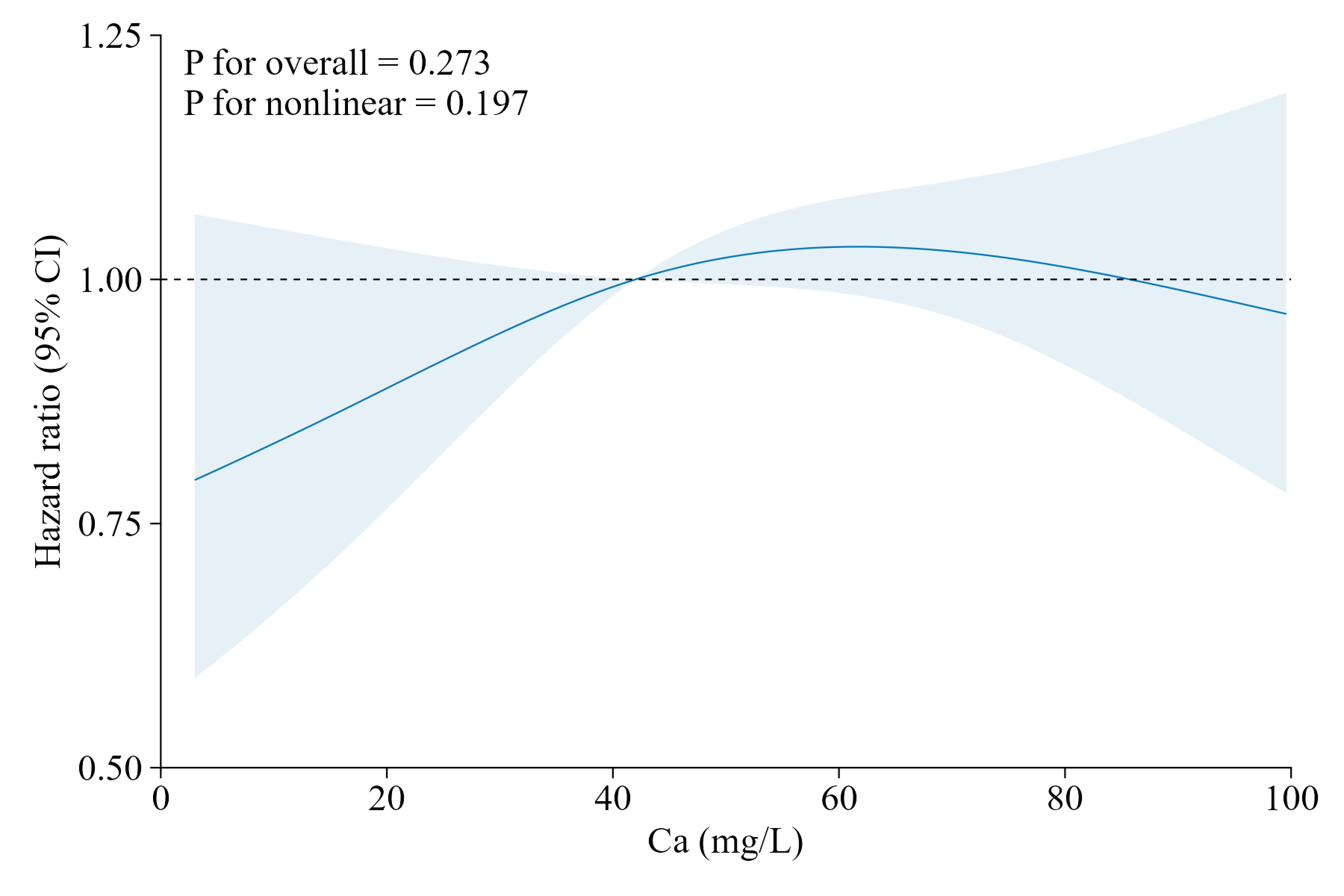

Supplement: S5 Fig — (TIF) [file pone.0326052.s016.tif]

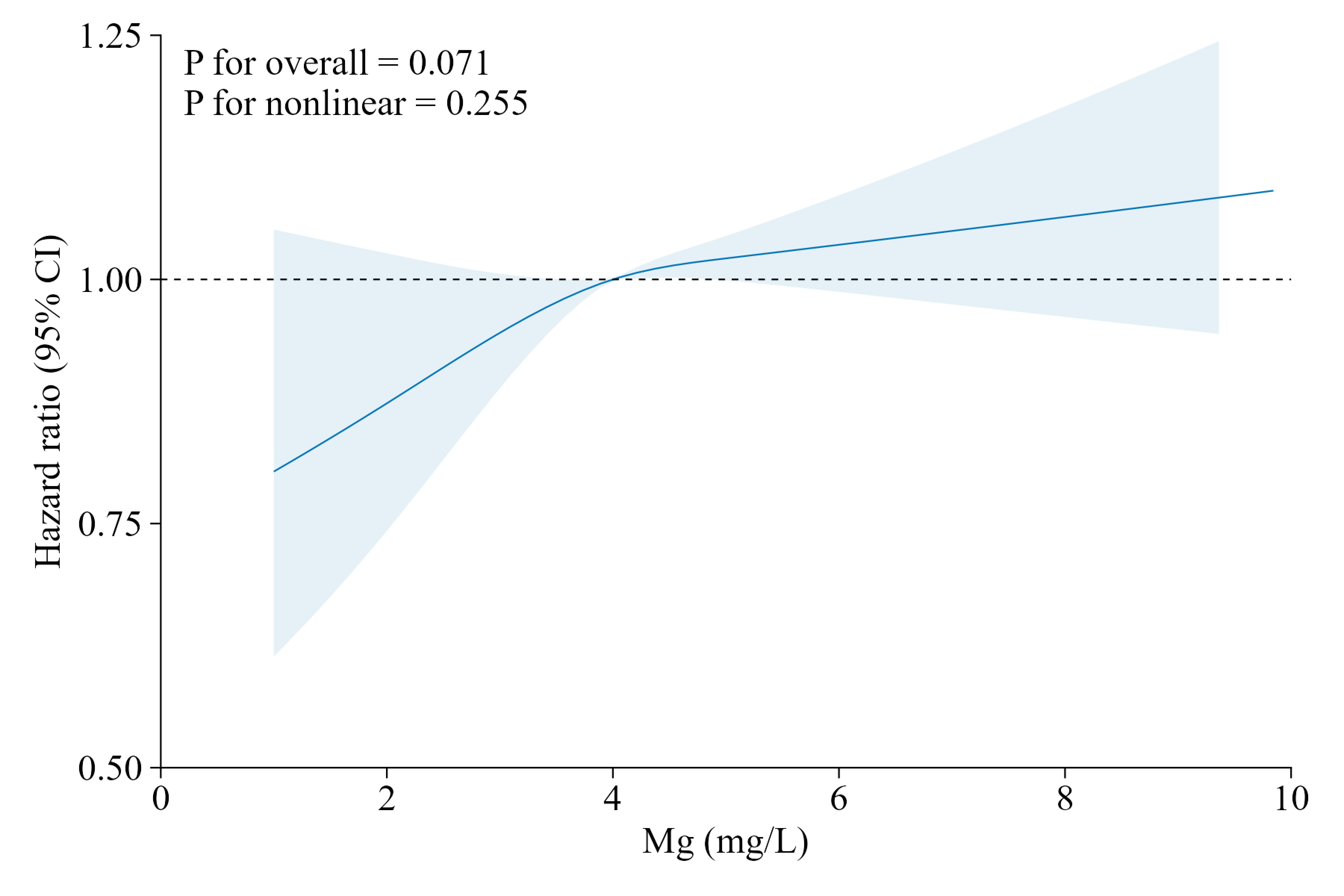

Supplement: S6 Fig — (TIF) [file pone.0326052.s017.tif]
